# Predicting clinical outcomes in a blended care intervention for early psychosis: Acceptance and Commitment Therapy in Daily-Life (ACT-DL)

**DOI:** 10.1038/s41398-024-03214-1

**Published:** 2025-01-07

**Authors:** Rafaël A. Bonnier, Joanne R. Beames, Glenn Kiekens, Evelyne van Aubel, Frederike Schirmbeck, Lieuwe de Haan, Machteld Marcelis, Mark van der Gaag, Ruud van Winkel, Therese van Amelsvoort, Thomas Vaessen, Ulrich Reininghaus, Ginette Lafit, Inez Myin-Germeys

**Affiliations:** 1 Department of Neurosciences, Research Group Psychiatry, Center for Contextual Psychiatry, KU Leuven, Leuven, Belgium; 2 Faculty of Psychology and Educational Sciences, KU Leuven, Leuven, Belgium; 3 Department of Medical and Clinical Psychology, Tilburg University, Tilburg, The Netherlands; 4 Department of Psychiatry, Amsterdam UMC, University of Amsterdam, Amsterdam, The Netherlands; 5 Department of Public Mental Health, Central Institute of Mental Health, Medical Faculty Mannheim, University of Heidelberg, Mannheim, Germany; 6 Department of Psychiatry and Neuropsychology, School for Mental Health and Neuroscience, Maastricht University, Maastricht, The Netherlands; 7 Institute for Mental Health Care Eindhoven (GGzE), Eindhoven, The Netherlands; 8 Department of Clinical Psychology, VU Amsterdam, Amsterdam, The Netherlands; 9 Department of Neurosciences, Psychiatry Research Group, Center for Clinical Psychiatry, KU Leuven, Leuven, Belgium; 10 Faculty of Behavioral, Management & Social Sciences, Department of Psychology, Health, and Technology, University of Twente, Enschede, The Netherlands; 11 Health Service and Population Research Department, Institute of Psychiatry, Psychology and Neuroscience, King’s College London, London, UK; 12 ESRC Centre for Society and Mental Health, King’s College London, London, UK

**Keywords:** Neuroscience, Psychology, Schizophrenia

## Abstract

ACT in Daily Life (ACT-DL) is a blended-care Ecological Momentary Intervention that extends ACT into the daily life of individuals, improving psychotic distress, negative symptoms, and global functioning. However, it remains unclear whether ACT-DL works equally for everyone. We investigated whether moderators (i.e., sociodemographic information, personality, and trauma history) determine clinical outcomes in individuals with early psychosis receiving ACT-DL. Seventy-one participants from the INTERACT trial, using ACT-DL, were analyzed. Outcomes included psychotic distress, negative symptoms, global functioning, and psychological flexibility. Using multivariate-multilevel models, we evaluated the effects of sociodemographics, personality, and childhood trauma across baseline, post-intervention, and six- and 12-month follow-ups. Sociodemographic characteristics and personality predicted clinical outcomes. Higher education demonstrated more substantial improvement in global functioning at 6- (*B* = 7.43, p = 0.04) and 12-FU (*B* = 10.74, p = 0.002) compared to lower education. Higher extraversion showed less improvement in negative symptoms at 12-FU (*B* = 1.24, p = 0.01) and more improvement in global functioning at post-intervention (*B* = 0.39, p = 0.046) and 6-FU (*B* = 1.40, p = 0.02) compared to lower extraversion. Higher negative affectivity showed more improvement in negative symptoms at 12-FU (*B* = −1.59, p = 0.001) and higher psychological flexibility at 12-FU (*B* = 8.38, p = 0.001) compared to lower negative affectivity. Our findings suggest that while ACT-DL improves clinical outcomes in individuals with early psychosis, the improvement rate is dissimilar for individuals and predictable by baseline characteristics. If replicated, these findings enable precision medicine approaches in allocating ACT-DL for early psychosis.

## Introduction

Psychosis exists on a continuum [1, 2], ranging from subclinical psychotic experiences in the Ultra-High-Risk stage (UHR) and First Episode Psychosis (FEP) to fully developed psychotic disorders like schizophrenia [3, 4]. This continuum includes individuals who may not meet the criteria for a clinical diagnosis of psychosis but who experience psychotic-like symptoms, distress, and impairment. Psychological interventions for early-stage psychosis are available and have shown promising results in reducing symptoms and distress, improving functionality, and preventing or delaying the onset of a full-blown psychotic disorder [5, 6]. Blended-care interventions combining digital tools with traditional face-to-face therapy have proven effective in various mental disorders [7–9]. However, it remains unclear what factors predict individual responses to blended-care interventions for early psychosis. This lack of knowledge limits the ability to personalize interventions and create appropriate clinical guidelines about what works for whom [6].

One promising example of a blended-care intervention for early psychosis is the Ecological Momentary Intervention (EMI) named “Acceptance and Commitment in Daily Life” (ACT-DL). ACT-DL combines face-to-face Acceptance and Commitment Therapy (ACT) with a smartphone application (app) that assesses momentary psychological states and provides users with ACT exercises and metaphors in their daily lives outside of the therapy room [10]. The efficacy of ACT-DL as an EMI was evaluated in a Randomized Controlled Trial (RCT) called INTERACT [11]. In the INTERACT trial, the ACT-DL intervention demonstrated improved psychotic distress compared to baseline, although no significant difference was found compared to treatment as usual (TAU). However, ACT-DL generally showed small to moderate effects in improving negative symptoms and global functioning compared to TAU [12]. While specific individuals improved, others may not have benefitted as much or may have potentially deteriorated. Moderator analysis is one way to explore individual differences in treatment response. Variables of interest are sociodemographic information, personality traits, and childhood trauma, which are rooted in their established relevance to psychological therapy outcomes and engagement, particularly within early psychosis populations.

Age, sex, and education level have been consistently highlighted in the literature as variables that shape not only the perception of psychological interventions, including EMI’s, but also the actual engagement with such treatments [13–15]. For instance, young individuals’ generally positive attitudes towards mobile health applications suggest a receptivity that might enhance intervention efficacy compared to older individuals [13, 15, 16]. Differences in sex might similarly affect ACT-DL’s efficacy as women are more likely to engage with apps designed to improve health, whereas young men tend to lose interest more quickly. Conversely, lower education levels have been linked to reduced engagement and poorer outcomes [14, 17, 18]. Thus, age, sex and education level might explain potential barriers that blended-care interventions must overcome to be universally effective. Second, personality traits have been studied in relation to first-episode psychosis (FEP) and offer a window into understanding individual psychological predispositions affecting psychotherapy outcomes [19–24]. Individuals with FEP reported lower levels of extraversion and higher levels of negative affectivity — defined as the inclination towards sociability and positivity, and frequent and intense experiences of negative emotions and interpersonal manifestations respectively— compared to healthy controls [25, 26]. These personality dimensions potentially affect ACT-DL’s efficacy as high-trait negative affectivity and low-trait extraversion are both associated with higher symptom severity at baseline, lower treatment adherence, and less therapeutic improvements [19]. Lastly, the inclusion of childhood trauma as a predictor is necessary because early adverse experiences can fundamentally alter the course and prognosis of FEP [27], with substantial evidence indicating that trauma history adversely affects treatment response [28, 29]. However, research specifically assessing the effects of sociodemographics, personality, and childhood trauma in the context of EMI’s (ACT-DL) is scarce, warranting additional investigation.

To better understand who might benefit most from ACT-DL, the current study aims to explore whether inter-individual factors moderate the effects of ACT-DL on psychotic distress, negative symptoms, global functioning, and psychological flexibility over time. We will investigate the following confirmatory hypotheses: (1) individuals with a lower education level will show less improvement in negative symptoms, psychotic distress, global functioning, and psychological flexibility than individuals with higher education levels over time; (2) individuals who score higher on negative affectivity or lower on extraversion will show less improvement over time in negative symptoms, psychotic distress, global functioning and psychological flexibility than individuals who score lower on negative affectivity or higher on extraversion; and (3) individuals with higher levels of childhood trauma will show less improvement over time in negative symptoms, psychotic distress, global functioning and psychological flexibility than individuals with lower levels of childhood trauma. Additionally, we will perform an exploratory analysis to investigate whether age and sex moderate the effect of ACT-DL on psychotic distress, negative symptoms, global functioning, and psychological flexibility over time. Findings will provide clinical guidance for whom ACT-DL might be an appropriate blended-care intervention. All statistical analyses were a-priori specified in a post-registered analysis plan (https://osf.io/xr9as/?view_only=a1ec33c65c824ca899aef08ffaec2bc6), which is a registration after data is collected but before data has been accessed and analyzed.

## Methods

### Participants

This study conducts secondary analyses of the INTERACT dataset (Reininghaus et al. [11]). The INTERACT dataset contains one hundred and forty-eight participants (78 UHR and 70 FEP) recruited from secondary mental health services in five regions in the Netherlands and Belgium. For inclusion, individuals needed to be between 15 and 65 years old, fluent in Dutch, and needed to provide written informed consent to be included. If UHR, participants could not use antipsychotic medication for psychotic symptoms before the study; if FEP, participants needed to have had one psychotic episode within the last three years. Exclusion criteria included a primary diagnosis of alcohol/substance abuse or dependence and severe endocrine, cardiovascular, or brain disease. The current study only included individuals randomized to the ACT-DL condition (*n* = 71). Refer to the protocol paper for more information on the sample and methods used in INTERACT [11].

### Procedures

Participants were recruited from secondary mental health services in five regions in the Netherlands and Belgium: (1) Amsterdam, (2) The Hague, (3) Maastricht/Eindhoven, (4) Vlaams-Brabant/Antwerpen and (5) Oost- & West-Vlaanderen. Participants were provided with initial information about the study by their treating clinician. The study team contacted individuals interested in participating, provided a complete explanation, and asked them to sign the informed consent form. When participants signed informed consent, the researcher conducted a full eligibility assessment. Eligible participants were randomly assigned in a 1:1 ratio to either the experimental condition (ACT-DL + TAU) or the control condition (TAU). Post-intervention and follow-up assessments were carried out by trained researchers who were blind to the participants’ assigned condition. Assessments were conducted at baseline, after the 8-week intervention period, and after six-month and 12-month follow-ups. The study received ethical approval from the MERC at Maastricht University Medical Centre (MUMC), the Netherlands (reference: NL46439.068.13) and the University Clinic Leuven, Belgium (reference: B322201629214).

### Intervention

The ACT-DL intervention [10, 30] consisted of eight weekly manualized ACT-sessions administered face-to-face by a trained clinician and an EMI in the form of an app. EMIs are interventions that are provided in the context of daily life [31]. ACT uses cognitive and behavioral techniques to improve psychological flexibility (i.e., adapting cognitive strategies to changing environmental conditions), decrease stress, and improve quality of life [32]. The first session involved general psychoeducation; the following six sessions focused on ACT skills, including acceptance, cognitive defusion, self-as-context, contact with the present moment, values, and committed action [32–34]. The final session was a summary of all the previous components. The EMI app prompted participants daily at eight semi-random moments for three days after each face-to-face session, starting from the second session onwards. Participants answered a brief questionnaire about their current mood, psychotic experiences, and activities, followed by an exercise or metaphor related to the ACT component covered in the previous session. Participants could also start ACT exercises from the app at any time on command. After the intervention period, access to the app was discontinued [11].

### Measures

#### Moderators

Demographic variables included sex (male or female), age in years, and education level. Education level was defined as the highest educational degree achieved and was divided into six categories: ‘elementary school,’ ‘secondary school – general education,’ ‘secondary school – technical education,’ ‘secondary school – vocational education,’ ‘Bachelor,’ and ‘Master.’ We redefined education level into two categories: ‘lower education,’ including elementary and all secondary school degrees, and ‘higher education,’ including Bachelor’s and Master’s degrees.

The personality traits extraversion and negative affectivity were assessed with The Eysenck Personality Questionnaire-Revised Short Form (EPQR-SF) [35, 36], a self-report questionnaire consisting of 48 items with a binary (YES or NO) answer option. Extraversion and negative affectivity were assessed using the 12 items of the corresponding subscales, with higher scores reflecting higher trait levels. The internal consistency of the extraversion and neuroticism subscales have been assessed in a reliability generalization study and were found to be very good to excellent, with (α = 0.82) and (α = 0.83), respectively [37]. In our sample, internal consistency was excellent for extraversion (α = 0.85) and neuroticism (α = 0.84).

Childhood trauma was measured using the Childhood Trauma Questionnaire Short Form (CTQ-SF) [38]. The CTQ-SF is a widely used 28-item self-report measure designed to assess the frequency and severity of childhood abuse and neglect, including emotional, physical, and sexual abuse and emotional and physical neglect. Items are rated on a 5-point Likert scale reflecting the frequency of the traumatic experience. Given that the different forms of trauma often occur together and strict separation of individual effects of a specific type is difficult [39–41], we computed a CTQ total score from the sum of all subscale scores (range: 25 to 125). We ran descriptive analyses using the describe function to visually check the variability of childhood trauma responses. Data were positively skewed, indicating that more individuals had lower trauma scores than individuals with high trauma. We used the categories’ no-low trauma’ (CTQ ≤ 51), ‘moderate trauma’ (51 < CTQ ≤ 68), and ‘severe trauma’ (CTQ > 68) based on cut-off scores of the individual trauma type subscales [38]. The internal consistency in our sample was excellent (α = 0.90).

#### Clinical outcomes

Psychotic distress was measured using the Comprehensive Assessment of At-Risk Mental States (CAARMS) positive symptom subscales [42]. This semi-structured interview assesses the intensity, frequency, and emotional distress of various positive symptoms to determine if an individual meets the criteria for UHR or FEP. The CAARMS has good psychometric properties and is widely adopted as a high-quality tool for assessing changes in psychotic distress in early psychosis [43, 44]. A psychotic distress total score was calculated by summing scores from the four CAARMS positive symptom scales (unusual thought disorder, non-bizarre ideas, perceptual abnormalities, and speech disorder), each ranging from 0 to 100, with 0 meaning ‘*not distressed at all’* and 100 meaning ‘*extremely distressed*.’ Missing distress scores were excluded from the calculation as they indicate a lack of distress for that symptom.

Negative symptoms were assessed with the Brief Negative Symptom Scale (BNSS) [45]. The BNSS captures the five negative symptoms reached by the NIMH consensus [46] (Avolition, Blunted affect, Anhedonia, Alogia, and Asociality) and measures an individual’s ability to experience normal distress (Distress subscale). These six subscales have 13 items each, rated using a 7-point Likert Scale ranging from 0 ‘*No impairme*nt’ to 6: ‘*Severe deficit’*. A total score was calculated by summing the subscale scores (range from 0 to 78), with higher scores indicating more severe negative symptoms. The BNSS has good convergent and discriminant validity, is sensitive to change, and has good internal consistency [47]. The within-person (ω = 0.90) and between-person (ω = 0.93) consistency was excellent in our sample.

Global functioning was assessed using the single-item Social and Occupational Functioning Assessment Scale (SOFAS) [48]. The SOFAS focuses exclusively on the individual’s social and occupational functioning level and is not directly influenced by the overall severity of the individual’s psychological symptoms. The SOFAS is rated on one continuous scale ranging from 1 ‘*Severe malfunctioning in a wide range of activities*’ to 100 ‘*Superior functioning in a wide range of activities’*.

#### Clinical process outcome

Psychological flexibility was measured using the Flexibiliteits Index Test (FIT-60), a reliable and validated self-report instrument sensitive to change [49, 50]. The FIT-60 comprises six subscales: ‘Acceptance,’ ‘Defusion,’ ‘Self as context,’ ‘Attention for here and now,’ ‘Values’, and ‘Commitment.’ Each subscale consists of 10 items scored on a 7-point Likert scale ranging from 0 ‘*Strongly disagree’* to 6 ‘*Strongly agree*’. A total score was calculated by summing the scores from the different skills, with higher scores meaning more psychological flexibility. Within-person and between-person consistency in our sample was excellent (ω = 0.92 and ω = 0.97, respectively).

## Data analysis

All statistical analyses were performed in R (version 4.2.2) and specified in the post-registered analysis plan (https://osf.io/dx2rp), which is a registration after data is collected but before data has been accessed and analyzed.

Analyses were conducted in R (version 4.2.2) according to intention to treat principles [12]. Given the hierarchical structure of the data, multilevel modeling was used to examine the effects of inter-individual factors on clinical outcomes over time. Multivariate multilevel models were estimated for the clinical outcomes (psychotic distress, negative symptoms, and global functioning), and univariate multilevel models were estimated for the clinical process outcome (psychological flexibility). Scores at post-intervention, six-month, and 12-month follow-up were the dependent variables. Separate models were estimated for each moderator (age, sex, education, negative affectivity, extraversion, childhood trauma), resulting in six multivariate and six univariate models. Time was added as a categorical moderator in each model with 0 = baseline, 1 = post-intervention, 2 = 6-month follow-up, and 3 = twelve-month follow-up. Age and sex were added as covariates in the models assessing the moderating effects of education level, personality traits, and childhood trauma. In other words, independent variables included moderator values, time, baseline scores (grand-mean centered), and the baseline score × moderator value × time interaction. Continuous moderators (age, extraversion, negative affectivity) were grand-mean centered to reduce multicollinearity and facilitate intercept interpretation. Categorial moderators were coded as follows: sex (0=male, 1=female), education level (0=lower education, 1=higher education), and childhood trauma (0=no-low trauma, 1=mild trauma, 2=high trauma). Models allowed for random intercepts and random slopes per timepoint. All models were estimated with the nmle package [51] using restricted maximum likelihood (REML) estimations. REML estimations allow for the use of all available data under the assumption that data are missing at random and that all variables associated with the missing values are included in the model. As established in the primary RCT analyses [12], there was no association between baseline variables and missingness of psychotic distress.

An omnibus-test of no difference was performed at all three time points (Wald-type test with df = 3 and α = 0.05) to determine whether each moderator had a significant overall effect on clinical outcomes. Time-specific contrasts were considered only if a moderator had a significant overall effect (each tested at α = 0.05). This approach precludes adjusting for multiple testing at the level of time-specific contrasts [12].

## Results

Table 1 presents the results of the omnibus-test of no difference (df = 3) for the overall effect of each moderator on the clinical outcomes. Table 2 shows descriptive statistics, adjusted mean differences with 95% confidence intervals, and p-values for moderators at each timepoint (post-intervention, six-month, and 12-month follow-up) compared to baseline. Results are interpreted in terms of an improvement in clinical outcomes.

**Table 1:** Moderators at baseline, Omnibus-test results.

				Omnibus-test^a^							
Moderator	Total (*N* = 71)			CAARMS		BNSS		SOFAS		Fit-60	
	*n*	%		χ²	p-value	χ²	*p*-value	χ²	*p*-value	χ²	*p*-value
Sex (Female)	52	59%		**11.52**	**0.009**	3.56	0.313	0.97	0.808	5.83	0.120
Education (Higher education)	27	38%		4.66	0.198	2.58	0.461	**10.20**	**0.017**	1.23	0.746
	Mean	SD	Range								
Age Male/Female	26/25.8	6.0	16−47	1.19	0.593	0.22	0.974	0.53	0.913	7.94	0.051
Neg. affectivity Male/Female	6.5/8.6	3.4	1−12	0.29	0.962	**11.28**	**0.010**	1.63	0.653	**14.22**	**0.003**
Extraversion	6.3/6	3.2	1−12	0.07	0.995	**15.09**	**0.002**	**10.06**	**0.018**	7.53	0.057
Moderate Childhood trauma	44/54	15.6	25−92	3.40	0.334	0.30	0.961	1.82	0.611	1.34	0.720
High Childhood Trauma				1.21	0.750	2.82	0.421	0.30	0.961	**7.88**	**0.049**

*CAARMS* Comprehensive Assessment of At Risk Mental State, *BNSS* Brief Negative Symptom Scale, *SOFAS* Social and Occupational Functioning Scale, *Fit-60* Flexibiliteits index test 60.

^a^Omnibus-test of no differences at all three timepoints (**χ**²(df) with 3 degrees of freedom), alpha = 0.05.

**Table 2:** Outcomes at baseline, post-intervention, and 6-month and 12-month follow-up.

Outcome					Adjusted mean differences at follow-up timepoints									
	Time	mean	*SD*	n^a^	Education					Sex				
CAARMS	Base	207.4	84.1	71	Value	*SE*	95%CI	t-value	*p*-value	Value	*SE*	95%CI	t-value	*p*-value
	Post	134.5	93.2	44	21.65	27.51	−32.27;75.58	0.79	0.432	−25.17	28.12	−80.29;29.95	−0.90	0.371
	FU6	106.0	75.1	35	17.58	25.95	−33.28;68.44	0.68	0.498	18.37	25.82	−32.24;68.98	0.71	0.477
	FU12	106.4	88.9	34	−39.35	24.63	−87.63;8.93	−1.60	0.111	−61.00	23.69	−107.43;−14.57	−2.58	**0.010**
BNSS	Base	17.2	11.9	71										
	Post	15.8	13.6	50	4.31	2.79	−1.16;9.77	1.54	0.123	5.22	3.24	−1.13;11.57	1.61	0.108
	FU6	11.0	9.1	41	2.97	3.24	−3.39;9.32	0.92	0.361	1.15	3.30	−5.32;7.62	0.35	0.728
	FU12	9.5	11.4	42	0.946	3.34	−5.60;7.48	0.28	0.783	3.73	2.94	−2.03;9.49	1.27	0.205
SOFAS	Base	40.0	11.1	71										
	Post	50.4	10.9	50	6.12	3.15	−0.06;12.29	1.94	0.053	−2.55	3.78	−9.96;4.86	−0.68	0.500
	FU6	57.7	10.5	41	7.43	3.62	0.34;14.52	2.06	**0.040**	0.06	3.84	−7.47;7.59	0.02	0.988
	FU12	57.9	12.2	41	10.74	3.45	3.98;17.49	3.12	**0.002**	−2.41	3.33	−8.94;4.12	−0.73	0.469
Fit-60	Base	171,1	52.7	66										
	Post	195.0	53.9	47	6.73	9.88	−35.64;5.76	−1.42	0.160	6.55	10.94	−14.89;27.99	0.60	0.550
	FU6	189.9	58.2	35	10.25	11.81	−22.67;18.17	−0.22	0.830	9.92	10.50	−10.66;30.50	0.95	0.347
	FU12	206.5	52.3	38	−6.91	14.898	−20.39;13.13	−0.42	0.672	5.32	8.87	−12.07;22.71	0.60	0.550

Outcome	Adjusted mean differences at follow-up timepoints															
	Time			Age					Negative affectivity					Extraversion		
CAARMS	Base	Value	*SE*	95%CI	t-value	*p*-value	Value	*SE*	95%CI	t-value	*p*-value	Value	*SE*	95%CI	t-value	*p*-value
	Post	2.09	2.47	−2.91;7.09	0.85	0.397	0.01	4.08	−7.90;8.10	0.02	0.981	0.21	5.63	−10.83;11.25	0.04	0.970
	FU6	−0.41	2.56	−5.41;4.59	−0.16	0.872	−0.63	4.18	−8.82;7.56	−0.15	0.888	7.29	5.65	−3.78;18.36	1.29	0.198
	FU12	−2.19	2.63	−7.19;2.81	−0.83	0.406	1.99	4.07	−5.99;9.97	0.49	0.632	0.64	5.91	−10.94;12.22	0.11	0.913
BNSS	Base															
	Post	−0.10	0,29	−0.67;0.47	−0.33	0.742	−0.56	0.43	−1.40;0.28	−1.29	0.197	0.79	0.45	−0.10;1.68	1.74	0.080
	FU6	0.03	0,31	−0.58;0.64	0.10	0.922	−0.79	0.50	−1.77;0.19	−1.58	0.114	0.15	0.52	−0.88;1.17	0.28	0.779
	FU12	−0.06	0.26	−0.57;0.45	−0.21	0.831	−1.59	0.49	−2.51;−0.61	−3.28	**0.001**	1.24	0.48	0.29;2.19	2.57	**0.011**
SOFAS	Base															
	Post	−0.24	0.33	−0.89;0.41	−0.72	0.473	−0.48	0.59	−1.64;0.68	−0.82	0.421	0.39	0.53	−0.64;1.43	0.75	**0.046**
	FU6	−0.16	0.36	−0.87;0.55	−0.44	0.658	0.25	0.61	−0.95;1.45	0.406	0.698	1.40	0.59	0.26;2.54	2.38	**0.018**
	FU12	−0.15	0.29	−0.72;0.42	−0.54	0.593	−0.24	0.50	−0.51;1.45	−0.49	0.632	−0.05	0.61	−1.24;1.14	−0.08	0.934
Fit-60	Base															
	Post	0.39	0.95	−1.47;2.25	0.41	0.683	2.98	1.59	−0.14;6.10	1.87	0.064	−3.95	2.16	−8.18;0.28	−1.83	0.070
	FU6	0.01	0.92	−1.79;1.81	0.02	0.988	2.43	1.84	−1.15;6.02	1.32	0.189	−2.67	2.22	−7.02;1.68	−1.20	0.232
	FU12	0.23	0.75	−1.24;1.70	0.31	0.760	8.38	2.34	3.79;12.97	3.58	**0.001**	3.21	1.73	−0.18;6.60	−1.85	0.067

Outcome		Adjusted mean differences at follow-up timepoints									
	Time	Mild trauma					High trauma				
CAARMS	Base	Value	*SE*	95%CI	t-value	*p*-value	Value	*SE*	95%CI	t-value	*p*-value
	Post	−18.87	28.01	−73.77;36.03	−0.67	0.501	59.14	45.04	−29.14;147.42	−1.31	0.190
	FU6	9.80	27.37	−43.85;63.45	0.36	0.720	−17.67	47.25	−110.28;74.94	−0.37	0.709
	FU12	−54.93	28.93	−111.63;1.77	−1.90	0.058	−25.26	41.25	−106.11;55.59	−0.61	0.541
BNSS	Base										
	Post	4.07	3.48	−2.75;10.89	1.17	0.243	3.32	5.16	−6.52;13.16	0.64	0.521
	FU6	0.85	3.51	−6.03;7.73	0.24	0.809	−5.59	5.23	−15.43;4.25	−1.07	0.286
	FU12	−1.15	3.27	−7.56;5.26	−0.35	0.726	1.48	4.66	−8.36;11.32	0.32	0.751
SOFAS	Base										
	Post	6.73	4.14	−1.27;14.73	1.62	0.105	4.86	6.06	−6.72;16.44	0.80	0.423
	FU6	4.49	4.25	−3.51;12.49	1.06	0.291	3.05	6.22	−8.53;14.63	0.49	0.624
	FU12	3.81	3.84	−4.19;11.81	0.99	0.321	4.04	5.44	−6.62;14.70	0.74	0.459
Fit-60	Base										
	Post	−0.56	11.73	^_^23.47;22.35	−0.05	0.962	−22.94	17.17	−56.59;10.71	−1.34	0.184
	FU6	1.25	11.65	−21.66;24.16	0.11	0.915	−20.82	16.58	−53.32;11.68	−1.26	0.212
	FU12	−3.13	9.53	−21.81;15.55	−0.33	0.743	−8.09	13.90	−35.33;19.15	−0.58	0.562

Adjusted mean differences are based on a complete-case ITT analysis of significant moderators from the omnibus-test.

*CAARMS* Comprehensive Assessment of At Risk Mental State, *BNSS* Brief Negative Symptom Scale, *SOFAS* Social and Occupational Functioning Scale, *Fit-60* Flexibiliteits index test 60, *SD* Standard deviation, *CI* Confidence Interval, *SE* Standard error.

^a^Number of observations differ from the maximum when participants did not complete the measure.

### Sociodemographics

**Fig. 1:**
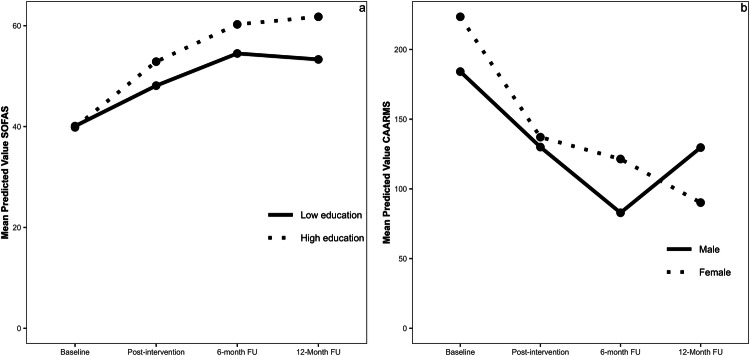
The effects of education level on Global Functioning (SOFAS), and of Sex on Psychotic Distress (CAARMS). **a** The effects of Education on Global Functioning (SOFAS), **b** The effects of Sex on Psychotic Distress (CAARMS).

The omnibus-tests investigating the effects of education level were significant for global functioning (**χ**² = 10.16, *p* = 0.017) but not the other clinical outcomes (**χ**²s ≤ 4.66, *p*s ≥ 0.198). Follow-up contrasts showed significantly greater improvement in global functioning at six-month follow-up (*B* = 7.43, SE = 3.62, *p* = 0.040) and 12-month follow-up (*B* = 10.74, SE = 3.45*, p* = .002) for individuals with high education compared to individuals with low education (Fig. 1a). There was a similar pattern at post-intervention, although the difference did not reach statistical significance (*B* = 6.12, SE = 3.15, *p* = 0.053).

For exploratory analyses, only the omnibus-test investigating the effects of sex on psychotic distress was significant (**χ**² = 11.52, *p* = 0.009). Follow-up contrasts showed significantly greater improvement for females compared to males in psychotic distress at 12-month follow-up (*B* = −61.00, SE = 23.69*, p* = 0.010) (Fig. 1b). There were no significant differences at any other time point.

### Personality traits

**Fig. 2:**
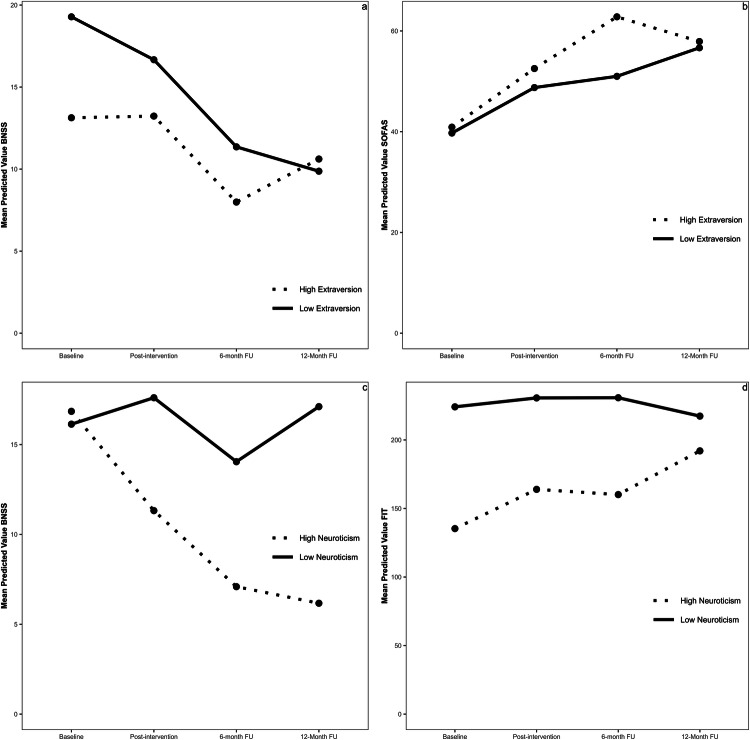
The effects of personality traits (extraversion and negative affectivity) on Negative Symptoms (BNSS), Global Functioning (SOFAS), and Psychological Flexibility (FIT). **a** The effects of Extraversion on Negative Symptoms (BNSS), **b** The effects of Extraversion on Global Functioning (SOFAS). **c** The effects of Negative affectivity on Negative symptoms (BNSS), **d** The effects of Negative affectivity on Psychological Flexibility (FIT). For the purpose of these plots we redefined the continuous variables Extraversion and Negative Affectivity as binary groups. The 25% lowest scores of Extraversion and Negative Affectivity were defined as the Low-groups, and the 25% highest scores of Extraversion and Negative Affectivity were defined as the High-groups.

The omnibus-tests investigating the effects of extraversion were significant for negative symptoms (**χ**² = 15.09, *p* = 0.002) and global functioning (**χ**² = 10.06, *p* = 0.018) but not the other clinical outcomes (**χ**²s ≤ 7.53, *p*s ≥ 0.057). For negative symptoms, follow-up contrasts did not show a significant difference at post-intervention (*B* = 0.79, SE = 0.45, *p* = 0.080) and six-month follow-up (*B* = 0.15, SE = 0.52, *p* = 0.799), but people with lower compared to higher trait extraversion showed a greater improvement at 12-month follow-up (*B* = 1.24, SE = 0.48, *p* = 0.011) (Fig. 2a). For global functioning, there was a significantly greater improvement for people with higher compared to lower trait extraversion at post-intervention (*B* = 0.39, SE = 0.53, *p* = 0.046) and six-month follow-up (*B* = 1.40, SE = 0.59, *p* = 0.018), but this was not retained at the follow-up assessments (*p* = 0.933) (Fig. 2b).

The omnibus-tests investigating the effects of negative affectivity were significant for negative symptoms (**χ**² = 11,28, *p* = 0.010) and psychological flexibility (**χ**² = 14.22, *p* = 0.003). Follow-up contrasts showed significantly greater improvement in negative symptoms (*B* = −1.59, SE = 0.49*, p* = 0.001) and psychological flexibility (*B* = 8.38, *SE* = 2.43*, p* = 0.001) at 12-month follow-up for people with higher compared to lower trait negative affectivity (Fig. 2c, d). There were no significant differences at any other time point.

### Childhood trauma

**Fig. 3:**
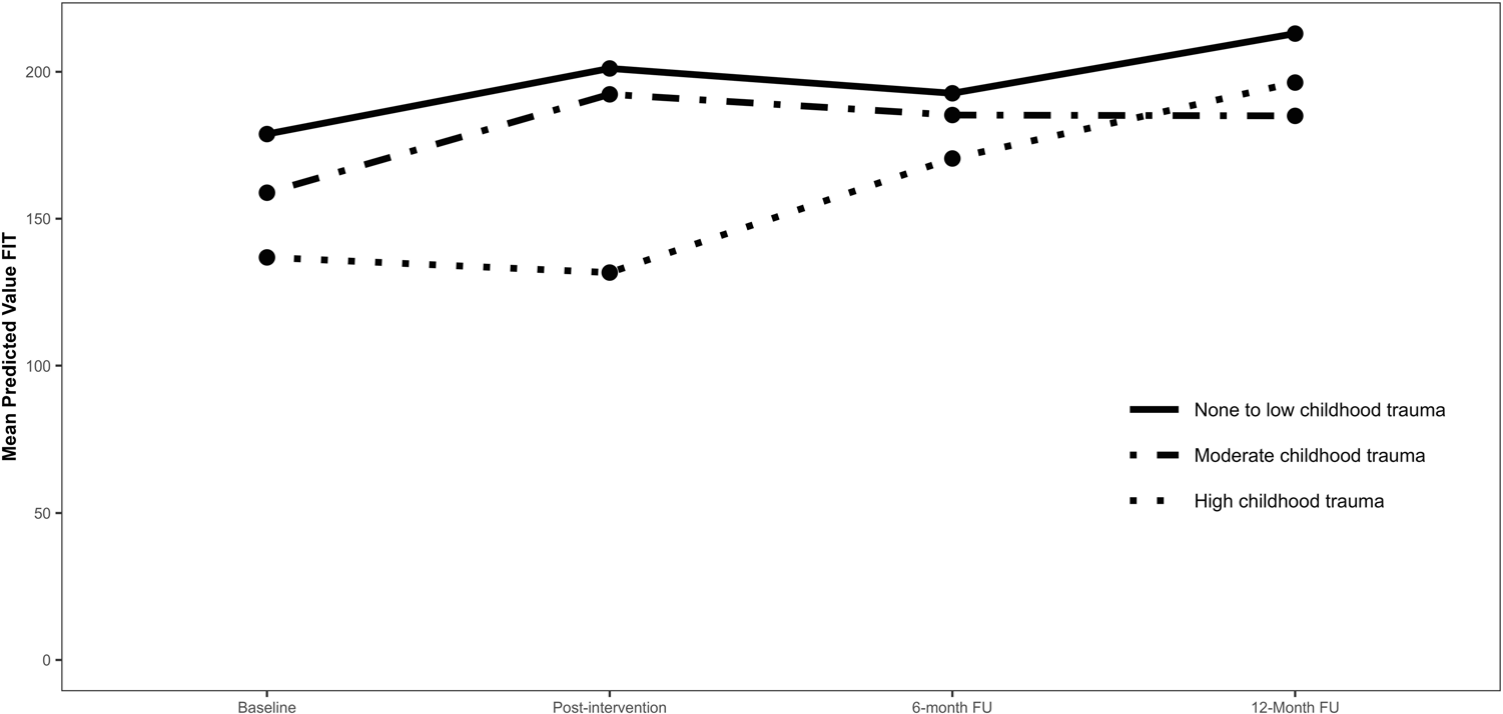
The effects of Childhood Trauma on Psychological Flexibility (FIT).

The omnibus-tests investigating the effects of childhood trauma were significant only for individuals with high childhood trauma compared to none-to-low childhood trauma on psychological flexibility (**χ**² = 7.88, *p* = 0.049). However, follow-up contrasts did not show significant differences at any time point (*p*s ≥ 0.184) (Fig. 3).

## Discussion

The present study examined if inter-individual factors moderated treatment responses in individuals experiencing early-stage psychosis who utilized the blended-care intervention ACT-DL [10]. We found that none of the moderators we examined negatively affected the effect of ACT-DL on clinical outcomes, meaning that ACT-DL may be an appropriate intervention for a wide range of individuals with early psychosis. While ACT-DL was generally effective, some moderators significantly influenced the improvement rate, with the most reliable effects for education and personality traits. Importantly, most of the significant effects were observed at the 6 and 12-month follow-ups, raising the possibility that our results may reflect differences in retention rates rather than immediate treatment responses.

For demographics, individuals with a higher educational background consistently showed more significant improvement in global functioning than those with a lower educational background over time. This result partially confirms our hypothesis that individuals with higher education would benefit more from ACT-DL, and converges with prior research on the effects of education on clinical outcomes in early psychosis [52, 53]. It is possible that the retained benefits in global functioning over time can be explained by other unobserved variables related to education level [54]. For example, socioeconomic status, better cognitive functioning, and baseline symptom severity might have influenced completion of between-session homework activities or ability to implement learned skills during moments of need. Recognizing the importance of equitable health outcomes, we see a need for future research exploring adaptations that could enhance the intervention’s efficacy for those with lower educational backgrounds. Identifying traits of lower-educated individuals who benefit from the intervention could inform these adaptations, aiming for inclusivity and optimal health outcomes for all participants. Moreover, recent qualitative findings of a pilot study testing an EMA tool by de Thurah et al. [55, 56] shows that additional therapist support and help in interpreting the EMA data is key to maximizing the benefits of EMA (and EMI’s). Furthermore, focusing on clear and concise instructions and metaphors might increase usability of the app overall. Our exploratory analyses showed that being female was associated with a significant decrease in psychotic distress at 12month FU. However, given that sex did not significantly affect any other outcome, we should conclude that ACT-DL generally works equally well for males and females. Lastly, age did not significantly affect improvement rates.

For personality traits, our results revealed that both extraversion and negative affectivity impacted responses to ACT-DL, albeit not always in keeping with our hypotheses. Consistent with our expectations, individuals with higher trait extraversion initially experienced a more rapid enhancement in their global functioning. However, at 12-month follow-up, higher and lower trait extraversion individuals converged to similar levels of global functioning. Contrary to our expectations, we found that individuals with lower trait extraversion and higher trait negative affectivity significantly improved negative symptoms and psychological flexibility. The pattern of results showed a steady decline in negative symptoms for individuals with low trait extraversion and high trait negative affectivity over time, alongside a steady increase in psychological flexibility, whereas the comparison groups exhibited stable symptom levels from baseline to 12-month follow-up. These effects could be explained by the possibility that individuals with lower extraversion or higher negative affectivity begin treatment with more severe symptoms and lower psychological skills, allowing them more room for growth. Prior research supports this idea, linking lower extraversion to decreased social skills and higher negative affectivity to more severe negative symptoms [19, 57]. A potential regression to the mean due to a broader margin for improvement could offer one explanation for these observations.

Additionally, it is worth considering that individuals with high trait negative affectivity, defined as frequent and intense experiences of negative emotions and interpersonal manifestations, might experience greater benefits from ACT-DL. This is because ACT emphasizes accepting negative emotions and enhancing values-based behaviors, directly addressing these vulnerabilities [58]. Importantly, we found evidence that childhood trauma did not have any moderating effects on clinical outcomes, proving ACT-DL to be a safe and effective treatment for individuals with a history of childhood trauma.

Our study opens multiple avenues for future research. Given that only 59% of our participants completed the full intervention [12], methods to boost therapy engagement are pivotal to maximizing clinical outcomes. One possible solution is to provide personalized feedback on an individual’s ecological-momentary-assessment data (EMA) in an interactive way. For example, embedding an overview page within the app that provides weekly statistics on the user’s EMA data and ACT-exercises might increase engagement with the intervention. Prior research supports this idea and found that personalized feedback is a strong motivator for using EMA tools and enhances uptake and adherence without altering the intervention’s core principles [59–61].

Additionally, integrating the self-monitoring data in individuals regular treatment, in preparation or during individual treatment sessions, might allow participants to gain more insight into their mental health, and how it relates to their daily lives [62, 63]. Another solution is to enhance ACT-DL’s efficacy at the individual level. Bacon et al. (2014) conducted qualitative research in which participants identified the distinct benefits of various ACT components. Mindfulness and Defusion, Acceptance, and Values were linked to stress reduction, providing life direction and meaning, and mitigating distress from unwanted private events, respectively. These findings suggest that different ACT components uniquely impact distinct facets of an individual’s mental health. Future research should explore whether specific ACT-DL components distinctly influence particular symptoms or psychological processes. Such understanding would allow therapists to tailor the ACT-DL intervention to the individual’s unique problem areas (e.g., psychotic distress).

## Limitations and future research

While our study provides valuable insights into the effects of moderators on treatment outcomes, several limitations warrant consideration. First, this study aimed to determine if specific moderators affected treatment outcomes in individuals who received ACT-DL. To maintain sufficient power for our confirmatory hypotheses, we narrowed our analysis exclusively to the ACT-DL group, refraining from comparisons with a control group. While we acknowledge the potential limitation of not having a control group, it is imperative to interpret our findings within the context of the primary outcome paper (Myin-Germeys et al. [12]), which found greater improvement in global functioning in negative symptoms for ACT-DL compared to TAU. These findings suggest that the observed effects may not solely be attributed to natural symptom dynamics or general response to interventions. However, future controlled studies conducted on larger samples are essential to validate our findings. Second, our sample comprised both FEP and UHR participants. While both categories signify early psychosis and are temporally and phenomenologically continuous, they represent different illness stages. Given that individuals with FEP had symptoms that reached the criteria for formal diagnosis and UHR did not, it is possible that both groups responded differently to ACT-DL. However, subgroup analysis in the primary study revealed no discernible differences between the UHR and FEP groups, and due to the study’s randomization [11, 12], these and other potential confounding variables were evenly distributed across conditions. Another potential cofounding variable might be the kind of treatment individuals received after the intervention was completed. For example, FEP individuals may have been prescribed antipsychotics throughout the trial. While this was also true for the control condition, and even though significant differences in treatment outcomes were observed between the ACT-DL and control groups [12], it remains unknown whether the longer-term effects identified in this study can be attributed exclusively to the ACT-DL intervention. In conclusion, this study highlights the importance of inter-individual factors as moderators of ACT-DL on clinical outcomes. Responses to ACT-DL were generally promising.

These limitations notwithstanding, this study highlights the importance of inter-individual factors as moderators of ACT-DL on clinical outcomes. We found evidence that education level, negative affectivity, and extraversion predicted improvement rates of ACT-DL in individuals with early psychosis. Future research should replicate these findings in more extensive and diverse samples and explore strategies to enhance the effectiveness of ACT-DL for a broader range of individuals.

## Data Availability

Deidentified data are available upon request through a data access system, Data Curation for Open Science (DROPS), administered via REDCap at the Center for Contextual Psychiatry, KU Leuven. Interested researchers can submit an abstract, which is subject to review by the research team to ensure there is no overlap with existing projects. Following abstract approval, a variable access request is submitted, and researchers are required to preregister their analysis plan. A dataset containing only variables required for the proposed analysis is then released to the researchers by a data manager, along with a time- and date-stamped receipt of data access.
